# Social and Professional Impact of Learning Communities Within the Alliances for Graduate Education and the Professoriate Program at Michigan State University

**DOI:** 10.3389/fpsyg.2021.734414

**Published:** 2021-11-25

**Authors:** Steven D. Thomas, Abdifatah Ali, Karl Alcover, Dukernse Augustin, Neco Wilson

**Affiliations:** ^1^The Graduate School, Michigan State University, East Lansing, MI, United States; ^2^Carlson School of Management, University of Minnesota, Minneapolis, MN, United States; ^3^Independent Researcher, Bethesda, MD, United States

**Keywords:** professional identity, learning community, diversity, minority student, graduate education, career readiness, STEM workforce, graduate student capital

## Abstract

At Michigan State University (MSU), the AGEP learning community features the participation of over 70% of the African-American, Latinx, and Native-American under-represented minorities (URM), also referred to as Black, Indigenous, and People of Color (BIPOC) doctoral students in fields sponsored by the National Science Foundation (NSF). Monthly learning community (LC) meetings allow AGEP participants to create dialogues across disciplines through informal oral presentations about current research. The learning communities also offer opportunities to share key information regarding graduate school success and experience; thus providing a social network that extends beyond the academic setting. At MSU, AGEP also provides an interdisciplinary and multigenerational environment that includes graduate students, faculty members, post-docs and prospective graduate students. Using monthly surveys over a 4-year period, we evaluated the impact of this AGEP initiative focusing on the utility of the program, perceptions of departmental climate, career plans and institutional support. Findings indicate that AGEP participants consider their experiences in the program as vital elements in the development of their professional identity, psychological safety, and career readiness. Experiences that were identified included networking across departments, focus on career placement, involvement in minority recruitment and professional development opportunities. Additionally, AGEP community participants resonated with the “sense of community” that is at the core of the MSU AGEP program legacy. In this article, we proposed a variation of Tomlinson’s Graduate Student Capital model to describe the AGEP participants’ perceptions and experiences in MSU AGEP. Within this 4-year period, we report over 70% graduation rate (completing with advanced degrees). More than half of Ph.D. students and almost 30% of master’s degree students decided to pursue academia as their careers. In addition, we found a high satisfaction rate of AGEP among the participants. Our analysis on graduate student capital helped us identify motivating capital development by years spent at MSU and as an AGEP member. These findings may provide some insight into which capitals may be deemed important for students relative to their experiences at MSU and in AGEP and how their priorities change as they transition toward graduation.

## Introduction

The Alliances for Graduate Education and the Professoriate (AGEP) program at MSU was seeded with funding from the National Science Foundation (NSF). The overarching goal of AGEP is to produce a national professoriate that reflects the diversity of the domestic population. A key and unique feature of the MSU program is the diverse AGEP Learning Community. Graduate students, post-docs, prospective graduate students and faculty who participate in the MSU AGEP Learning Community seek to help contribute to transforming the culture of United States colleges and universities to embrace building world-class STEM and the social, behavioral and economic sciences (SBE) faculties who fully reflect the diversity in race, gender, culture, and intellectual talent of the United States population. The MSU AGEP program was a part of the former Michigan AGEP Alliance (MAA), a consortium of five public universities: Michigan State University, the University of Michigan, Western Michigan University, Wayne State University, and Michigan Technological University.

What makes the MSU AGEP program unique are opportunities for information sharing, career skill-building, and leadership opportunities for participants to actively contribute to the success of the program. Lynch et al. have shown that a multi-prog approach like the MSU AGEP program are useful retention strategies (Lynch and Kathy, 2011). In response to a lack of diversity of faculty in United States universities, the goal of the MSU AGEP program is to aid in the recruitment and retention of graduate students and postdoctoral associates (or “post-docs”) from historically under-presented groups. Specific strategies used to summarize MSU AGEP program activities include: Community Building, Science Advocacy, Science Literacy, Outreach, and Leadership. The sense of community can be observed at the monthly MSU AGEP Learning Community (LC) meetings which fosters a multidisciplinary community of graduate students, post-docs, faculty members and undergraduate students.

In-person attendance ranges from 40 to 60 students per meeting. There were typically 11 LC meetings **per year** (2014–2018) between September and May. The meetings allow AGEP attendees to engage across disciplines while sharing “best practices” for succeeding in graduate school. Recurring activities with MSU AGEP LCs include the Student Chalk Talk presentations, faculty panels about academic careers, alumni panels about job searching, community acknowledgments, and networking discussion about student success strategies, career planning and science advocacy. Featured aspects of the community are cross-disciplinary discussions of a presenter’s research, called CrossTalks. An important hallmark of the MSU AGEP LC meetings were the interdisciplinary discussions and inter-generational conversation among students of different stages, faculty and invited undergraduates. Graduate student and post-doc recruitment for MSU AGEP meetings involved campus welcome events, national conference recruitment and presentations during faculty staff meetings.

Over the years, the AGEP Learning Community has developed into a model scholarly community, stimulating academic interests, promoting professional development, and cross-generational interactions among the students and participant alumni. Activities related to science advocacy include interactions with policy makers, science literacy through the annual AGEP Science Today Bulletin, outreach through cross-generational mentoring with MSU SROP students and leadership through active student engagement on the AGEP Student Steering Committee and during the annual Fall AGEP conference hosted by Michigan State University AGEP program. Student AGEP participants not only receive information, but they also contribute their expertise and expressions of graduate capital to the AGEP Learning community. We seek to use a proposed variation of Tomlinson’s model of graduate capital as a framework to describe our observations of open-ended responses to participant perceptions of their experiences of the MSU AGEP program. Our evaluation hopes to contribute to existing literature on peer-mentoring communities and professional identity formation within graduate education (Kim-Prieto et al., 2013; Russell et al., 2018).

The scope of this paper is to describe an exploratory study we conducted while analyzing survey data collected from 2014 to 2018 about how students perceive their engagement, learning outcomes, and application of knowledge based on their interaction with the MSU AGEP community. We also included our survey questions about their satisfaction within their home departments and future plans. We examined if there any differences in the responses among BIPOC and non-BIPOC attendees, as well as gender, STEM/social science degrees and years in AGEP and MSU. Over this 4-year period, we report a high graduation rate of AGEP community members. Our hope is that using the model of Graduate Student Capital and learning and environment measures will help to describe the reasons for these outcomes.

## Background and Relevant Literature

### Under-Representation in Academia and Its Ramifications

The low proportional representation of BIPOC scholars in faculty positions in the United States, jeopardizes the nation’s ability to innovate and address current global challenges (Hong and Page, 2004; U.S. Department of Commerce, National Economic Council, 2012; Freeman and Hrabowski, 2014). The contemporary composition of faculty demographics creates a barrier for the recruitment of BIPOC graduate students (Austin, 2002). Even when recruitment efforts have taken form, retention is still an issue among BIPOC graduate students and junior faculty due their elevated experiences of discrimination, marginalization and isolation, and impostor syndrome in comparison to their white counterparts (Gibbs and Griffin, 2013; Gibbs et al., 2014). In response, organizations such as the Alfred P. Sloan Foundation, NSF, and NIH have established programs to increase URM students’ access to advanced degrees in STEM disciplines. Examples of scholarship and capacity-building programs developed by NSF and NIH include the NSF’s Alliances for Graduate Education and the Professoriate (AGEP) and the Louis Stokes Alliance for Minority Participation (LSAMP), as well as NIH’s MARC U STAR and Bridges to the Doctorate (R25).

### Professional Identity Formation During Graduate School

Sutherland et al. (2010) explained that professional identity is one’s identity related to their professional roles and status. Berkenkotter et al. (1988) described the life of graduate students as that of becoming initiated into a research community through scholarly reading and writing practices, through interactions with faculty and peers as well as exposure to research methodology.

Ducheny et al. (1997) suggested that graduate student professional identity development typically includes three primary elements: (a) the importance of continued training and familiarity with relevant research, (b) the influence of a supportive peer group or mentor, and (c) the organization of professional development into stages articulated by formative events and level of training. Geraniou (2010) describes the life cycle of graduate students into three distinct stages, Adjustment, Expertise and Articulation. Geraniou describes the Adjustment stage as the natural process of coming to terms with what a Ph.D. degree is like and adjusting to its nature. The Expertise stage is articulated as applying background knowledge to solve the research problem. The third stage, Articulation Stage, involves the writing down the results in the form of a thesis/dissertation.

Gazzola et al. (2011) investigated what experiences and conditions counseling psychology doctoral students perceive as contributing to their professional identities. Their reported results showed that the following hindered students’ professional identity development: experiencing negative views of the profession, disappointment with institutional training, and internal conflicts (i.e., concerns about completing their graduate program). Gazzola et al. also reported, in contrast, positive experiences with clients during clinical training and achievements in the program confirmed their views of their professional identity.

The Tomlinson model is based on internal resources an individual has within the five dimensions of self (Tomlinson, 2017). These include **Human** (Gary Becker, 1993), **Identity** (Giddens, 1991; Beck and Beck-Gernsheim, 2002) **Cultural** (Bathmaker et al., 2013) (Burke, 2015), **Social** (Bourdieu, 1986), and **Psycho-social** (Brown et al., 2012). His model suggests that these forms of internal capital are acquired through graduates’ formal and informal experiences. We used a modified version of the Tomlinson model as a framework for analysis of our open-ended responses.

This paper attempts to modify the Tomlinson model somewhat and relabel Human capital as **Technical capital**, the development of specific discipline skills. In the context of our program, Technical capital is most often expressed in the technical chalk presentations. We also relabeled, Tomlinson original “Identity capital” as **Career Identity capital** but in agreement with Tomlinson original definition as the development of personal employment narrative.

Furthermore, we define **Cultural capital** as cultural confidence and desire to seek professional camaraderie with students/professionals of color. Tomlinson, originally conceived this as the formation of culturally valued knowledge, dispositions and behaviors that are aligned to the workplaces that graduates seek to enter.

Tomilson defines **Social capital** as relationships and networks that help mobilize graduates’ existing human capital and bring them closer to the labor market. Tomilson defines Psychological capital as the psychosocial resources which enable graduates to adapt and respond proactively to inevitable career challenges.

**FIGURE 1:**
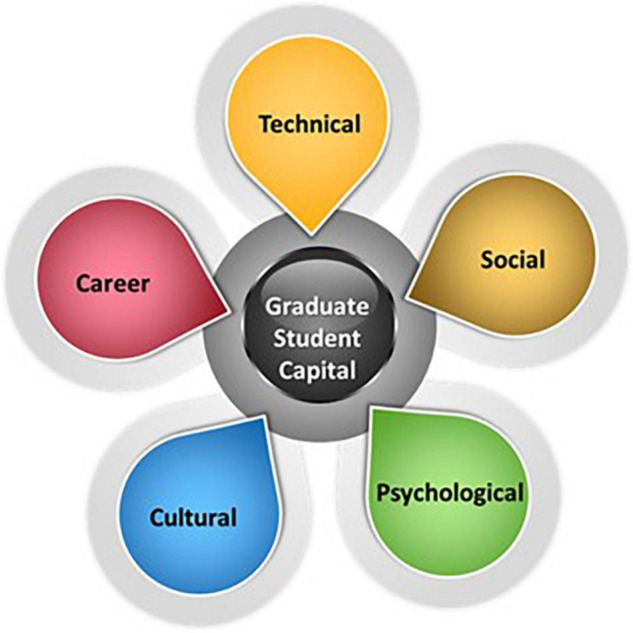
Proposed variation of graduate student capital model.

Our interest in adapting individual elements of this model can be further substantiated by other researchers for each of our proposed dimensions of Technical (building technical skills) (Ann et al., 2009; Choe and Borrego, 2020), Social (peer-mentoring and networking) (Tull et al., 2012; Bottoms et al., 2013; Montgomery, 2017; Williams, 2018), Psychological (psychological safety) (Lyman et al., 2020; Soares and Lopes, 2020), Cultural (cultural resilience) (Espino et al., 2010; Julia et al., 2020), Career Identity (socialization within the profession) (Kim et al., 2018; Bentley et al., 2019), see Figure 1.

### The Value of Peer Mentoring and Learning Communities in Graduate Education

Improving the mentoring relationship between faculty and their proteges has been proposed by many scholars to increase the academic success, self-confidence and motivation of graduate students (Komarraju et al., 2010). However, a growing number of scholars are also investigating the role fellow graduate students have on the academic and professional training of their peers as well as their socialization within the profession (Vosloo et al., 2014). Watson et al. (2009) study, they found that 46% of graduate students described peer mentoring as equally, if not more, effective than professional mentoring from faculty. However, only 25% of all individuals surveyed in the Watson et al. (2009) report indicated that a formal or informal peer mentoring program was in place within their academic program. Though institutions at the department level offer formal and informal programs for the development of future faculty, many are purely focused on the development of specific skills like teaching or the development of teaching philosophy and portfolios (Viall et al., 2008). Many students have shown that for BIPOC students, learning communities that address both the professional skill building as well as their unique experiences associated with their personal identity, can lead to higher retention rates (Tull et al., 2012; Drane et al., 2019).

## Materials and Methods

### Michigan State University Alliances for Graduate Education and the Professoriate Learning Community Demographics

The demographic information was obtained from AGEP community graduate students through their student records: gender, ethnicity, incoming year, graduation year (if applicable), and department affiliation. Demographic information from the AGEP community attendees (*N* = 299) was taken from members who attended at least one AGEP community meeting from January 2014 to May 2018.

Students who enrolled in graduate programs within the College of Natural Science, Engineering, Human Medicine and Veterinary Medicine were classified as **“STEM**.**”** Students who enrolled in graduate programs in the Chicano/Latino Studies or African and African-American Studies Program, College of Social Science and some selected programs in the College of Communication Arts and Sciences were designated as **“SBE”**. Other students, mostly those enrolled in the Colleges of Education and Music, were classified as **“Other**.**”** Collectively, the categories of SBE and Other will be combined as non-STEM for statistical analysis.

Completion results were based on the number of AGEP participants who graduated with a degree (Masters or Doctoral) by 2020. Students who graduated by December 2020 were categorized as **“Alumni**,**”** those who were still enrolled as **“Current**.**” Current participants** were divided into two groups based on their degree program, **Masters** or **Doctoral**. Participants who had not been enrolled since Fall 2020 and had not graduated, were designated as **“No-Degree**.**”** Students who left the university because they were denied graduation or dropped out are also included in this category. Students that self-identify as Black, Indigenous, and People of Color, they will be collectively referred to as BIPOC. White, non-Hispanic students will be referred to as White or Non-BIPOC.

### Program Evaluation and Survey

Program evaluations were distributed through monthly paper surveys. Participants were instructed to fill out the surveys once per semester. Even though responses were anonymous, participants were asked to enter the last 4-digits of the student ID number to monitor duplicates. For statistical analysis only one entry per student was used (first observation, *N* = 155). We did not use data from multiple observations from the person but plan those for future studies.

With our survey instrument, we investigated how students were interacting within the community (community interaction), which aspects of the program they found most important (important aspects), what strategies they learned (important strategies), how they perceived the larger MSU environment especially their home department (MSU Environment), as well as their future plans. Dependent variables we sought to determine any influence included race, gender, time in MSU AGEP, time at MSU, and major (STEM vs. non-STEM). We chose to look at the differences between years in AGEP at MSU versus years at MSU in general in order to examine if there differences in student responses based on their time on campus in comparison to their time within the MSU AGEP program itself.

Other questions that were asked but not analyzed in this report include their involvement in the AGEP community, stress coping strategies as well as current academic milestones (passed comprehensive exams, etc.).

#### Alliances for Graduate Education and the Professoriate Community Interaction

The following 4-item measure was developed to capture *Community Interaction*: (1) My participation has helped me attain my educational goals; (2) I encourage more students to participate in AGEP; (3) I feel more confident in my career because of AGEP; (4) I have an opportunity to learn from other graduate students. All items were assessed using a 5-point Likert-type scale (from 1 = “Strongly disagree” to 5 = “Strongly agree”).

#### Student Perceptions of Important Aspects of the Alliances for Graduate Education and the Professoriate Program

Responses to the open-ended question of “What is one of the most important aspects of the MSU AGEP Learning Community?” were coded based on the Graduate Student Capital model (Figure 1).

Below is a list of some of the qualitative responses from students based on the open-ended question of “What is one of the most important aspects of the MSU AGEP Learning Community?”

##### Technical

“Opportunity to present work and receive feedback.”

“The opportunity to present my research to a diverse audience.”

“Opportunity to hear interdisciplinary research, learn new concepts and see intellectual presentations.”

##### Social

“Opportunity to engage with students from across the U [University] (Appreciate that community members are welcome to bring their kids).”

“Building community with other like-minded students.”

“The friendship that I have formed, I use the community as support both academically and non-academically.”

##### Psychological

“Network, knowing that we are all in the struggle. Not feel alone.”

“Community, comfortability, access, belonging.”

“Unity, involvement and support.”

##### Cultural

“Being around scholars of color and across disciplines.”

“I think it’s the ability to discuss issues of diversity openly. There are a number of social issues we discuss in meetings and it’s always okay for people to address issues of underrepresented populations.”

“Interacting with a truly diverse community of scientists.”

##### Career

“Exposure to work in other disciplines and not only at the doctoral level. Access to role models in higher ed and administration and faculty.”

“Getting experiences socializing into academia.”

“Learning about opportunities for professional development, research funding, and post-doc information.”

#### Student Perceptions of Important Strategies of the Alliances for Graduate Education and the Professoriate Program

Responses to the open-ended question of “What is one of the most important strategies you have learned from AGEP meetings?” were coded based on the Graduate Student Capital model (Figure 1).

Below is a list of some of the qualitative responses from students based on the open-ended question of “What is one of the most important strategies you have learned from AGEP meetings?”

##### Technical

“How to talk about my research to a broad audience.”

“How to present and collect research and facilitate meeting and group talks.”

“Using visuals to represent concepts that may be unfamiliar to people outside of your disciplines.”

##### Social

“Strategies for networking outside of my department and college.”

“The importance and necessity of making connections w/fellow students. There connection provide interesting discussion topics and interdisciplinary perspectives to my work.”

“I liked the opening prompts that got us started at our tables.”

##### Psychological

“Have a community of support – seek out help if needed.”

“Talking across disciplines for advice and support.”

“Conflict resolution.”

##### Cultural

“To truly be a part of something bigger than yourself.”

“Initiating conversations with people in a diverse setting.”

##### Career

“A multiple approach of building your CV.”

“Seek resources within the graduate school.”

“How to negotiate effectively for an academic position.”

#### Michigan State University Environment Satisfaction

An eight item measure was developed to capture *MSU environment satisfaction*. Sample items for this measure include, “I am confident I will complete my degree,” “I am satisfied with my research project,” and “I am satisfied with the professional development I am receiving within my department.” All items were assessed using a 5-point Likert-type scale (from 1 = “Strongly disagree” to 5 = “Strongly agree”). Alpha or internal reliability for this measure was 0.79. For all analyses, a factor score consisting of all eight items was used.

1. I am confident I will complete my degree.
2. I am satisfied with my choice to come to MSU.
3. I am satisfied with my faculty advisor choice.
4. I am satisfied with my faculty committee choice.
5. I am satisfied with my research project.
6. I feel my undergraduate experience prepared me well.
7. I am satisfied with the social climate within my department.
8. I am satisfied with the professional development I am receiving within my department.

#### Future Plans of Participants

The future plans of the participants were assessed by asking them to check all that apply from a range of options that are listed below. Majority of the current participants selected (1) becoming a faculty member or (2) working in industry as part of their future plans. These two response options were used for subsequent analysis as outcome variables in examining their correlations with our main demographic variables.

1. Work in industry.
2. Work in a government lab or agency.
3. Go to professional school.
4. Become a faculty a member.
5. Start a business or become an entrepreneur.
6. Enter the military.
7. Teach at K-12 Schools.
8. Become a post-doc.
9. Other (specific here).
10. Undecided.

### Statistical Analysis

Our analytical approach included examining the associations between our focal independent variables along with several key outcomes of interest. To determine group differences, we used Fisher’s exact test for categorical variables, Kruskal–Wallis test for categorical and continuous variables, and Pearson’s correlation for two continuous variables. *P*-values of <0.05 were considered statistically significant. We also conducted moderated linear regressions with ordinary least square (OLS) as the estimation method. All moderation analyses were performed using the SPSS software. For each outcome (e.g., MSU environment satisfaction), the main effects and the interaction term were entered simultaneously. Significant interactive effects were further probed by creating a graph that illustrates the nature of the interaction. Lastly, simple slopes analyses were conducted for significant interaction terms.

## Results

### Michigan State University Alliances for Graduate Education and the Professoriate Placement Data

Of the **299** LC attendees, **241** are now listed as alumni who completed an advanced degree and **58** are still completing their degrees as of December 2020. Other community members included **4** completed their post-docs, 10 left with no-degree, and 1 deceased member. From the **241** alumni, **203** completed a doctoral degree and **38** completed a masters degree. From the doctoral alumni pool, **53.7%** are in academic positions, **20.2%** are in the private sector, **13.0**% are in other and **12.8%** have unknown placement. Within the masters pool, **28.9%** are in academic positions, **31.6%** are in the private sector, **23.7%** are in other, and **15.7%** have unknown placement. The category of “other” for career placement is defined for AGEP alumni working in government, non-profit, independent contractor, or K-12 education. From the **4** post-doc alumni, **2** are in academic positions. See Table 1 for MSU AGEP participant demographics for January 2014-May 2018. See Tables 2, 3 for breakdown of MSU AGEP alumni by demographics and job placement respectively as of December 2020.

**TABLE 1:** Demographics of MSU Alliances for Graduate Education and the Professoriate (AGEP) learning community attendees from 2014 to 2018.

	STEM	SBE	Other	BIPOC	White	Male	Female
Doctoral	81	116	55	231	21	81	171
Masters	18	15	10	40	3	17	26
Post-doc	4	0	0	4	0	0	4

**TABLE 2:** Demographics of MSU Alliances for Graduate Education and the Professoriate (AGEP) learning community demographics of 2014–2018 alumni who completed degrees.

	STEM	SBE	Other	BIPOC	White	Male	Female
Doctoral	62	95	46	188	15	67	136
Masters	17	14	7	35	3	16	22

**TABLE 3:** Job placement of MSU AGEP learning community alumni as of December 2020.

	Academia	Private-sector	Other-sector	Unknown
Doctoral stem	18	24	8	12
Masters stem	5	4	4	4
Doctoral non-stem	91	17	19	14
Masters non-stem	6	8	5	2

### Alliances for Graduate Education and the Professoriate Community Interaction

Table 4 shows the overall percentage of responders that said “Agree” or “Strongly Agree” for the Community Interaction measure. We find that over time students increase their agree/strongly agree ratings (not shown in tables). When analyzing these trends using years at MSU and years in AGEP as variables, we calculated correlations for MSU years (0.3220) and AGEP years (0.2753) with respective *p*-values at 0.0002 and 0.0015.

**TABLE 4:** Community interaction results.

My participation has helped me attain my educational goals	86.6%
I encourage more students to participate in AGEP	98.1%
I feel more confident in my career because of AGEP	86.1%
I have an opportunity to learn from other graduate students	100.0%

### Student Perceptions of Important Aspects of the Program

Here, we examined how students at different stages of their graduate career and years of participation in MSU AGEP program (as measured in years at MSU and years in AGEP, respectively) expressed their varied learning outcomes from their AGEP community engagement. A summary table of each of the coded 155 responses are in Table 5. We statistically compared years in AGEP/MSU to their coded qualitative responses (see Table 6). We found no significant differences between groups.

**TABLE 5:** Summary of coded responses to important aspects and strategies open-ended questions.

	Aspects	Strategies
Technical	44	56
Social	35	21
Career	17	28
Psychological	26	12
Cultural	23	2
No response	10	36

**TABLE 6:** Response analysis from important aspects open-ended question.

	Aspects					
	
	Career	Cultural	Psychological	Social	Technical	*p*-value
**Years at MSU**						
Median, IQR	2 (2−4)	4 (1−5)	1.5 (1−4)	1 (2−4)	2.5 (1−4)	0.340
**Years in AGEP**						
Median, IQR	1 (1−3)	2 (1−5)	1.5 (1−2.5)	1 (0.5−4)	2 (0−3)	0.331

### Student Perceptions of Important Strategies Learned

We also examined how students at different stages of their graduate career and years of participation in MSU AGEP program (as measured in years at MSU and years in AGEP, respectively) expressed their varied learning outcomes from their AGEP community engagement. A summary table of each of the coded 155 responses are in Table 5. We statistically compared years in AGEP/MSU to their coded qualitative responses (see Table 7). We found statistically significant associations between graduate career stages and learning outcomes from their AGEP community engagement.

**TABLE 7:** Response analysis from important strategies open-ended question.

	Strategies					
	
	Career	Cultural	Psychological	Social	Technical	*p*-value
**Years at MSU**						
Median, IQR	1.5 (1−3)	2.5 (2−3)	2 (1−3)	2 (1−4)	3 (2−5)	0.017
**Years in AGEP**						
Median, IQR	1 (0−3)	3 (1−5)	2 (1−2)	1 (0.5−3)	3 (1−4)	0.044

### Student Responses to Michigan State University Environment Satisfaction Questions

**FIGURE 2:**
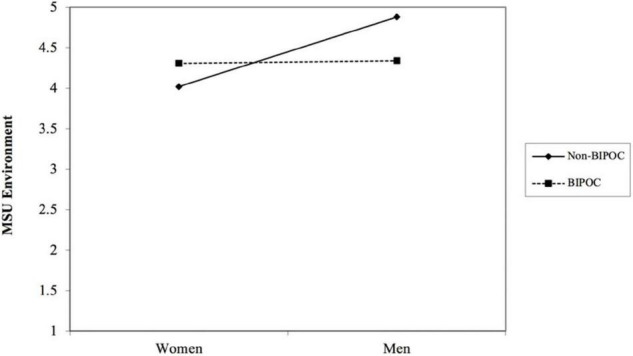
Gender interacting with BIPOC to predict MSU environment (overall measure).

**FIGURE 3:**
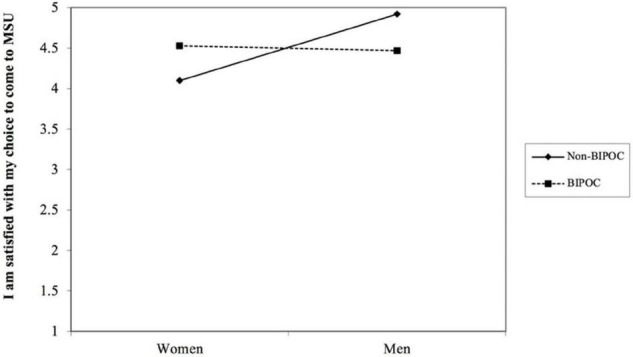
Gender interacting with BIPOC to predict MSU environment item “I am satisfied with my choice to come to MSU.”

**FIGURE 4:**
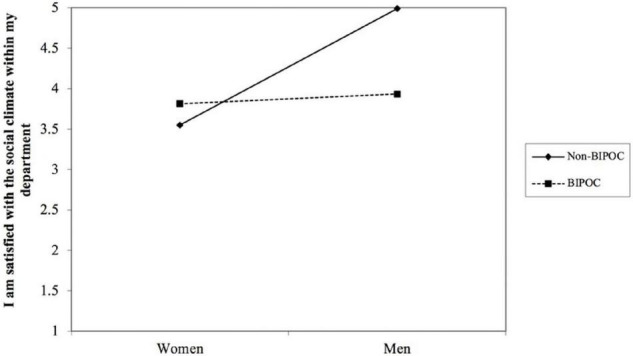
Gender interacting with BIPOC to predict MSU environment item “I am satisfied with the social climate within my department.”

**FIGURE 5:**
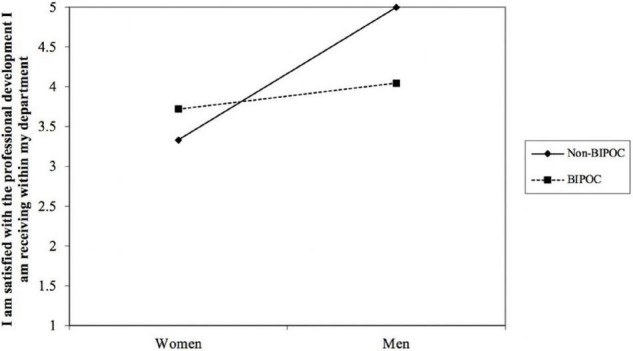
Gender interacting with BIPOC to predict MSU environment item “I am satisfied with the professional development I am receiving within my department.”

Presented in Table 8 are the regression results for the outcome variable of MSU environment satisfaction. We found that gender interacted with BIPOC status to predict MSU environment satisfaction (*b* = −0.83, *p* < 0.05) (see Table 8 and Figure 2). Simple slopes analysis indicated that the effect of gender on MSU environment satisfaction was significant for non-BIPOC AGEP members (*b* = 0.86, *p* < 0.05), as compared to BIPOC members (*b* = 0.03, *ns*). To further probe the MSU environment satisfaction measure, we did separate analyses on the item level to determine which items were contributing to the observed differences. Table 8 shows that of the eight total items that make up the MSU environment satisfaction measure, only three items were significant and reproduced similar results as seen in the overall measure. These items were: “I am satisfied with my choice to come to MSU,” “I am satisfied with the social climate within my department,” and “I am satisfied with the professional development I am receiving within my department.” Figures 2–5 shows the pattern of the interaction effect, which are also consistent with an interaction effect found for the overall MSU environment measure.

**TABLE 8:** Gender interacting with BIPOC to predict MSU environment satisfaction questions.

Predictor	MSU environment		I am satisfied		I am satisfied		I am satisfied with the	
	satisfaction		with my choice to		with the social climate		professional development I am	
	(overall measure)		come to MSU		within my department		receiving within my department	
	Beta^a^	SE^b^	β	SE	β	SE	β	SE
Intercept	4.02		4.18		3.56		3.33	
Gender^c^	0.86**	0.38	0.82*	0.44	1.44**	0.69	1.67***	0.68
BIPOC^d^	0.29	0.19	0.43**	0.22	0.26	0.38	0.39	0.39
Gender X BIPOC	−0.83**	0.39	−0.88*	0.46	−1.32*	0.75	−1.34*	0.75
*F*-Statistic	1.93		1.66		1.52		2.37	

*N = 118;*

^a^Unstandardized beta coefficient; ^b^Standard error; ^c^Gender coded (0 = females, 1 = males); ^d^BIPOC coded (0 = Non-BIPOC, 1 = BIPOC). *p < 0.10, **p < 0.05, ***p < 0.01.

### Future Plans of Alliances for Graduate Education and the Professoriate Participants

Presented in Table 9 are the inter-correlations among the focal variables in the study. Gender, STEM versus non-STEM, years in AGEP, and years at MSU were significant correlates of the two outcome variables of interest: Plans to become a faculty member and working in industry.

**TABLE 9:** Correlations between demographics and future plans variables.

Variables	Future plans: Become	Future plans: Work
	faculty member	in industry
Gender^a^	0.21**	0.05
BIPOC^b^	0.17	–0.03
STEM^c^	−0.31***	0.52***
Years in AGEP	0.18**	0.03
Years at MSU	0.20**	−0.15*

*N = 134; ^a^Gender coded (0 = females, 1 = males); ^b^BIPOC coded (0 = Non-BIPOC, 1 = BIPOC); ^c^STEM coded (0 = Non-STEM, 1 = STEM).*

*p < 0.10,**p < 0.05, ***p < 0.01.

## Discussion

In this article, we proposed a variation of Tomlinson’s Graduate Student Capital model to describe the AGEP participants’ perceptions and experiences in the MSU AGEP program and presented the findings of our 2014–2018 survey of MSU AGEP participants. Within this 4-year period, we report over 70% graduation rate (completing with advanced degrees). More than half of Ph.D. students and almost 30% of master’s degree students decided to pursue academia as their careers. In addition, we found a high satisfaction rate of AGEP among the participants. Our analysis on graduate student capital helped us identify motivating capital development by years spent at MSU and as an AGEP member. These findings may provide some insight into which capitals may be deemed important for students relative to their experiences at MSU and in AGEP and how their priorities change as they transition toward graduation. Our initial findings show that students report that the strategies learned within the MSU AGEP community vary at slightly different rates across their years in AGEP and MSU. Furthermore, we also see that environmental factors become salient when we consider both gender and race together instead of analyzing them separately.

Additionally, we did not include the entire dataset since there were multiple observations from the same participant. We plan to conduct longitudinal studies on the dataset in the future that will take into account the repeated measurements presented in the data.

### Michigan State University Alliances for Graduate Education and the Professoriate Placement Data

In this study, we examined the placement rate of our MSU AGEP alumni who attended Learning Community meetings from January 2014 to May 2018, we have shown a high retention rate of our attendees leaving MSU with an advanced degree. Over 50% of our doctoral members are working in academia and almost 30% masters student alumni are well. We also find that our STEM students are open to a wider variety of job sectors due to their high demand skill sets. This is in alignment with our survey results related to their future plans.

### Alliances for Graduate Education and the Professoriate Community Interaction

When we asked about how the AGEP program helped with their goals, confidence and career, over 80% of respondents stated that they agree or strongly agree. Over the years, we received a higher percentage of “agree” or “strongly agree” responses.

### Student Perceptions of Important Aspects of Program

All five aspects of the adapted Tomlinson model were present in how the attendees operationalized their experiences in AGEP and warrants further investigation into their perceptions of their employability (Table 6).

### Student Perceptions of Important Strategies Learned

We see from Table 7 that first year graduate students (years at MSU) and first year AGEP attendees (years at AGEP) are self-identifying different strategies they are learning in comparison to their third year counterparts. For example, first-years are gravitating toward building their career identity capital (identifying resources and socializing within their desired career sector), while third-year students are focusing on their technical skill building capital (presentation skills). Students within their second year begin to value the strategies related to psychological safety, peer-mentoring and cultural resilience.

### Student Responses to Michigan State University Environment Satisfaction Questions

Our findings about gender and race are consistent with other researchers (Ellis, 2001; Cortland and Kinias, 2019). Cortland and Kinias (2019) observed that women in the workforce feel less work satisfaction when they feel less role models, sponsors, or peer support are available. Ellis investigated the experiences of black and white doctoral students at a predominantly white research institution to determine whether there were differences in student socialization, satisfaction with doctoral study, and commitment to degree completion based on race or gender. Overall, Ellis reported that women of color were negatively affected the most. Our findings about departmental level professional development dissatisfaction are also consistent with other scholars (Williams, 2002). Williams examined the perceptions of the amount and types of social support reported by BIPOC and White doctoral students during graduate school. White doctoral students reported greater program satisfaction, more positive perceptions of the academic environment, and fewer program problems than Black doctoral students. Black doctoral students reported more negative perceptions of the social environment than the other group in the Williams study.

### Closing Thoughts

Our program evaluation hopes to contribute to existing literature on peer-mentoring communities and professional identity formation within graduate education (Trede, 2012). Insight into professional identity formation can be helpful in improving the education of advanced degree earners (Cruess et al., 2015). Our application of a graduate student capital model can be used as a framework to describe student experiences/needs using affirming vocabulary versus deficit models when examining and implementing minority student-centered programming and workforce development.

## Data Availability Statement

The raw data supporting the conclusions of this article will be made available by the authors, without undue reservation.

## Ethics Statement

The studies involving human participants were reviewed and approved by the Michigan State University. Written informed consent for participation was not required for this study in accordance with the National Legislation and the Institutional Requirements.

## Author Contributions

ST supervised the project and wrote the manuscript with support from AA, KA, DA, and NW. ST, AA, and KA verified the analytical methods. DA and NW cleaned and prepared the data for analysis. All authors contributed to the article and approved the submitted version.

## Conflict of Interest

The authors declare that the research was conducted in the absence of any commercial or financial relationships that could be construed as a potential conflict of interest.

## Publisher’s Note

All claims expressed in this article are solely those of the authors and do not necessarily represent those of their affiliated organizations, or those of the publisher, the editors and the reviewers. Any product that may be evaluated in this article, or claim that may be made by its manufacturer, is not guaranteed or endorsed by the publisher.
